# Nutritional Status of Children with Attention Deficit Hyperactivity Disorder: A Pilot Study

**DOI:** 10.1155/2010/767318

**Published:** 2010-06-28

**Authors:** Joy Y. Kiddie, Margaret D. Weiss, David D. Kitts, Ryna Levy-Milne, Michael B. Wasdell

**Affiliations:** ^1^Food Nutrition and Health, University of British Columbia, Vancouver, BC, Canada V6T 1Z4; ^2^Division of Child Psychiatry, University of British Columbia, Vancouver, BC, Canada V6T 2A1; ^3^Children's and Women's Health Centre of British Columbia, P.O. Box 178, P2-229, Vancouver, BC, Canada V6H 3N1; ^4^Oncology Nutrition, BC Cancer Agency, Vancouver, BC, Canada; ^5^Fraser Health Authority, British Columbia, BC, Canada

## Abstract

*Objectives*. This is a pilot study of the dietary intake and nutrient status of children with Attention Deficit Hyperactivity Disorder (ADHD). *Method*. Nutritional assessment of 43 children aged 6–12 with ADHD was performed using a 3-day food record, 24-hour recall, and serum assessors. *Results*. Macronutrient intake and consumption of Low-Nutrient Foods were comparable to population norms; however, 66% were found to be deficient in zinc and 23% in copper. *Conclusions*. This pilot study reports the food intake and nutrient status of children with ADHD and shows a predisposition for low zinc and copper status in ADHD.

## 1. Introduction

While diet and supplements in Attention Deficit Hyperactivity Disorder (ADHD) have and continue to remain a popular explanation of the disorder that has generated a wealth of research [1–4], there has been minimal empirical research characterizing both dietary intake and serum nutrients. Children with ADHD may be at risk for a variety of nutrient deficiencies due to the attentional demands required to sit through a meal to obtain adequate levels of nutrient intake, as well as the appetite suppressant effects of treatment medication. 

There are currently only four studies assessing dietary intake in ADHD; a study of ADHD preschoolers [5], two overseas studies assessing dietary intake in school-aged children in Taiwan [6] and Poland [7], and one study assessing dietary intake in the United States [8]. Methodological limitations in former research include the use of dietary assessment tools which were not validated for pediatric use [8] and a comparison of grouped dietary intake data on ADHD children to assessors of individual dietary requirements, rather than assessors of group dietary requirements [8, 9]. 

There is now evidence to suggest that ADHD may be associated with low trace mineral status, specifically zinc and iron [10, 11]. This finding is of considerable interest given the fact that iron and zinc, as well as copper, are essential cofactors in the production of dopamine and norepinephrine; two neurotransmitters that play an essential role in the etiology of ADHD [12–14]. 

Serum zinc levels have been found to be significantly lower in ADHD children compared to controls in several controlled studies around the world, including Poland [15], Turkey [7], Israel [16], the United States [8, 17], and the United Kingdom [18], despite the fact that the diet of these countries is very different, suggesting this to be a finding worth further investigation. Robust correlation has also been found between serum zinc and attention ratings, but not hyperactivity [8]. Two controlled trials in ADHD showed a benefit with dietary zinc supplementation [19] of Events Relate Potential (ERP), a measured brain response that is directly the result of a thought or perception [20]. 

Several authors have also found an association between low serum ferritin and ADHD [11, 21, 22] with low serum ferritin being correlated with more severe ADHD symptoms [23–25]. In iron supplementation studies of ADHD children with low serum ferritin (i.e., <30 *μ*g/L), significant improvement in ADHD symptoms was reported in ADHD children who received iron over a placebo [26]. 

The objective of this pilot study was to describe the dietary food intake and assess the nutrient status of DSM-IV diagnosed ADHD children aged 6–12 years. We hypothesized that children with ADHD have abnormal dietary intake and low micronutrient status.

## 2. Method

### 2.1. Ethics Approval

The University of British Columbia Clinical Research Ethics Board and the British Columbia Children's and Women's Hospital Research Committee approved the study protocol.

### 2.2. Sample

Sequential patients at the Provincial ADHD Program in Vancouver, Canada were offered participation in the current study and 44/46 accepted. All parents gave informed consent, and children completed assent forms; the study was then carried out in accordance with the Declaration of Helsinki, revised. Eighteen (18) stimulant-treated, 9 atomoxetine-treated, and 17 treatment-naïve children aged 6–12 years participated. Inclusion criteria were a primary diagnosis of ADHD and proficiency in English. Those patients on ADHD medication had to have been stable on the current type and dose for at least 6 months and had to be taking medication 7 days a week. Exclusion criteria were the use of any additional medication known to alter food intake, other than stimulants or atomoxetine prescribed for ADHD, or any medical condition that could alter nutritional status. Female subjects had to be premenarchal.

### 2.3. Assessment Methods

#### 2.3.1. Diagnosis of ADHD

Diagnosis was based on a two-hour clinical interview and a semistructured diagnostic interview of childhood psychiatric disorders; the Kiddie Schedule for Affective Disorders and Schizophrenia (KSADS-E) [27, 28]. ADHD symptom assessment was based on the parent and teacher completion of the Swanson Nolan and Pelham (SNAP-IV) scale which is an 18-item DSM-IV based ADHD checklist [29] and parent, teacher, and youth completion of the Strengths and Difficulties Questionnaire which includes a well-normed subscale for ADHD [30].

#### 2.3.2. Anthropometric Data Analyses

Height and weight of ADHD children were assessed using calibrated stadiometers and electronic scales and compared with standard growth charts from the Centre for Disease Control (CDC) [31].

#### 2.3.3. Blood Parameter Analyses

Nonfasting, venous blood samples were collected by a registered phlebotomist at the Outpatient Laboratory of British Columbia Children's Hospital. Trace mineral analysis was conducted by the Outpatient Laboratory at Children's Hospital and pyridoxine/B_6_ analysis was carried out by Hospitals in Common (HIC). Comparative data received from the hospital laboratory provided the normal ranges by age and gender.

#### 2.3.4. Nutritional Assessment Procedures

The 24-hour food recall was administered according to a multipass time interview format, the same method used in the NHANES comparative data [32]. Any supplementation with vitamins and/or minerals was recorded. Dietary intakes from the 24-hour food recall were used to determine the percentage of Low-Nutrient Density (LND) foods in the diet according to the same method as the comparative study [33]. 

The three-day food record data served as an approximation of usual intake and was used to determine the proportion of the group below the Estimated Average Requirement (EAR) for specific micronutrients [34]. Dietary intakes provided in the three-day food records were also used to determine the percentage of subjects below, within, and above the Acceptable Macronutrient Distribution Ranges (AMDR) for protein, fat, and carbohydrate [9]. Energy intake was compared to the Estimated Energy Requirement (EER) for those subjects based on age, gender, body weight in kilograms, height in meters, and a physical activity (PA) coefficient estimated from the interview of lifestyle and exercise habits and micronutrient intake was compared to the Estimated Average Requirements (EAR) for each subject using the “cut-point method”; [9, 35] the same method was used in comparative data [34]. 

A computer-generated randomization schedule of two weekdays and one weekend day was produced for up to 100 subjects and was utilized sequentially for all 43 subjects in this study in order to assign subjects to days in which to collect data for the three-day food record. 

Both parents of subjects and subjects were jointly instructed on portion estimation and provided with visual estimates to aid in three-day food record data collection [36]. 

All dietary intake data was entered into a computer nutrient database (Food Processor SQL 2005-06, ESHA Research, Salem, Oregon) modified to include the Canadian Nutrient File.

### 2.4. Data Analysis

All statistical comparisons were performed using two-tailed tests set at *P* < .05 level of significance. Chi-square tests were used to determine differences in categorical data between sample and populations. Independent sample *t*-tests were used to evaluate differences between groups of continuous, normally distributed variables. Comparisons of data obtained from the present study and previous population studies were conducted by first transforming the standard error given by population studies to a standard deviation statistic based on population size. Independent sample *t*-tests were then used to compare the results. As this study was conducted in Canada, local recent population dietary intake data were used as a basis for comparison [20, 37]. As no Canadian population nutritional biochemistry data are available, serum values were compared to the most recent available US population biochemical data (NHANES II) [38]. Since the study was exploratory, no Bonferroni correction for multiple analyses was performed.

## 3. Results

### 3.1. Demographic Characteristics of the Sample

This study was composed of 37 males (84%) and 7 females (16%) with a mean age of participants being 8.5 years. Children were pooled into two groups; aged 6–8 years (*n* = 23) and 9–12 years (*n* = 21). A compliance of 88.6 percent was obtained for the 3-day food record collection and 82% for the nutritional status analysis. The majority of children in the study were from two parent families (70.5%). The overall level of education of parents was high with 70% having obtained a trade or vocational certificate, a college certificate, or a university degree. The household gross income was greater than $60,000 per year for 61.4% of the families; with 27.3% of the families having an income greater than $100,000. Only two children were from families where the gross family income was less than $15,000.

### 3.2. Anthropometric Data

Twenty percent of children were above the 97th percentile for height-for-age and twenty percent of children were above the 90th percentile for weight-for-age. The mean height-for-age of drug-treated subjects (72.5 percentile, *n* = 27) was similar to the mean height-for-age of drug-naïve subjects (75.1 percentile, *n* = 17).

### 3.3. Dietary Supplement Usage

Majority of the child cohort (e.g., 54.5%) took a dietary supplement that consisted of vitamin-mineral combination containing iron, zinc, and copper (*n* = 14); a vitamin-mineral supplement with iron only (*n* = 3); and a vitamin-only supplement (*n* = 7). Subjects that took the vitamin-mineral supplement had similar serum zinc and copper concentrations (e.g., 10.9 *μ*mol/L ± 2.2 and 15.5 *μ*mol/L ± 2.1, resp.) compared to counterparts that did not consume a supplement. There was no significant difference in serum zinc and copper levels observed in our ADHD cohort and those obtained from laboratory norm values for children of similar age and gender without ADHD.

### 3.4. Macronutrient Intake

Estimated Energy Requirements (EER) were calculated on subject age, physical activity, and height and weight. Estimated daily energy intake was determined from a three-day food record (Table 1). Almost 50% of the children aged 6–8 years met their energy requirement (range = 51%–127% of EER). In comparison, 70% of children aged 9–12 years had EER that ranged from 62%–154%. Most of the children in the study were within the Acceptable Macronutrient Distribution Range (AMDR) for protein, carbohydrate, and fat. For example, 94% of children in the study had protein intakes within the acceptable 10%–30%, while 67% of children in the study had carbohydrate intakes that were within the 45%–65% of energy intake. Slightly more than half of children (55.6%) in the study were within the 25%–35% range of the AMDR for fat; whereas 38.9% were exceeding the AMDR for fat intake. There were no differences in the percent macronutrient distribution in diets of ADHD children compared to similar age-paired BC children surveyed in the Canadian Community Health Survey (CCHS) [24].

Approximately 60% of ADHD children aged 6–8 years followed in this study consumed less than 2 servings of Meat and Alternates per day. This result, when compared to a recent population data that indicated that Canadian children in this age group consume an average of 2.4 servings of Meat and Alternates per day [20], is relatively low and noteworthy since this food group is the major source of dietary iron, zinc, and copper [39]. Finally, there was also no significant difference in the percentage of low-nutrient density (LND) foods consumed by ADHD boys aged 8–12 years in the study (32.3%) when compared with boys of the same age from a US population study (28.6%) (*t* (17) = 0.77, *P* = .46) that used NHANES II data [33]. There were insufficient girls in the study to enable comparison with the population normal data.

### 3.5. Micronutrient Intake

In Table 2, we show that 28% of children aged 6–8 years did not meet the Estimated Average Requirement (EAR) for zinc or copper. In comparison, 61% and 39% of children aged 9–12 years were below the EAR for zinc and copper, respectively. The majority of children in both age groups met the EAR for Vitamin B_6_ and all children in the study met the EAR for iron.

Since there are no recent Canadian data available on mean dietary intakes of iron, zinc, and copper in children aged 6–12 years, data from this study was compared to the most recent US dietary intake data from NHANES III and is presented in Table 3. Dietary intakes of zinc, copper, and Vitamin B_6_ in ADHD children were significantly (*P* < .001) below the population norms, while no differences in iron intake were found.

### 3.6. Serum Micronutrient Levels

Serum zinc and copper concentrations of ADHD children examined in this study were found to be lower than the most recent US population data (NHANES II) for non-ADHD children aged 6–8 and 9–13 years (Table 4). For example, ADHD children aged 6–8 had serum zinc that was significantly lower than population normal data (*t* (34) = 2.48, *P* = .02), with 26% having serum zinc values below the 2.5th percentile of the NHANES II cutoffs for zinc deficiency for nonfasted individuals (≤10.1 *μ*mol/L/66 *μ*g/dL). The prevalence of zinc deficiency in ADHD children in this study was eight times greater than the normal prevalence of 3.3% reported for males and 3% for females [40]. Twenty percent of the ADHD children, aged 9–12, had serum zinc values below the 2.5th percentile cutoffs for zinc deficiency, a finding which is 20 times greater than the normal prevalence of zinc deficiency of 1% in females and slightly less than 1% in males. Compared with a 2005 study by Arnold of children aged 5–10 years, children in the current study in the same age range consumed half the levels of zinc (*t* (29) = 8.06, *P* < .001) and exhibited double the prevalence of low-serum zinc status (27.1%) [8].

Iron status and vitamin B_6_ status of all children in this study were within lab normal range.

Pearson correlations showed no relationship between dietary intake of zinc and serum zinc status (*r* (36) = −0.13, *P* = .22), but there was a significant relationship between dietary intake of copper and serum copper status (*r* (36) = 0.420, *P* < .01) in ADHD children. Finally, no significant differences were found in dietary macro- and micronutrient intake between drug-naïve and drug-treated participants when assessed in terms of total caloric intake. This corresponded with the fact that mean height-for-age for drug-treated subjects was similar to that for drug-naïve subjects (e.g., within the 72–75 percentile).

### 3.7. Relationship between Trace Mineral Status and Dietary Intake

Pearson correlations showed no relationship between dietary intake of zinc and serum zinc status (*r* (36) = −0.13, *P* = .22), but did show a relationship between dietary intake of copper and serum copper status (*r* (36) = 0.420, *P* < .01). Independent sample *t*-tests did not find a relationship between serum zinc status and meeting the recommendations of consumption from the Meat and Alternate food group (*t* (16) = 0.63, *P* = .54). 

 In subjects that took vitamin-mineral supplements, mean serum zinc and mean serum copper were 10.9 *μ*mol/L ± 2.2 and 15.5 *μ*mol/L ± 2.1. This compared well with subjects that did not take vitamin-mineral supplements (11.4 *μ*mol/L ± 1.3 for serum zinc and 15.4 *μ*mol/L ± 2.9 for serum copper). Among subjects that took vitamin-mineral supplements, one sample *t*-tests also showed no significant difference, between observed and lab normal values for serum zinc (*t* (23) = 1.54, *P* = .13), and copper (*t* (23) = 1.40, *P* = .17) values. 

There were no significant differences between drug-naïve and drug-treated participants in the dietary intake of protein (*t* (30) = 0.56, *P* = .58), carbohydrate (*t* (30) = 1.24, *P* = .23), and fat (*t* (30) = 0.36, *P* = .74) as assessed in calories per day or any difference in the mean dietary intake of calories (*t* (30) = 0.01, *P* = .94) between drug-naïve and drug-treated participants as determined by independent sample *t*-tests.

## 4. Discussion

The current study reports that ADHD children in this sample are taller and heavier than Canadian population norms based on standard CDC growth charts and taller but not heavier than what has been reported in previous ADHD samples [41, 42]. Our finding is consistent with previous reports which indicate that ADHD children in other local samples may be larger than non-ADHD children of the same age and gender [43]. It is unclear why ADHD children in these samples do not appear to follow standardized growth curves. 

Independent sample *t*-tests showed no significant difference in height-for-age (*t* (38) = 0.11, *P* = .91) between drug-treated subjects and drug-naïve subjects indicating that the medication status of the subjects examined in this study was not a factor. This may be because of the young age and low drug exposure of the children in the study. 

This study found no significant difference between the ADHD children in this sample and population norms in mean energy intake, calories or proportion of calories derived from protein, fat, or carbohydrate as compared with data from the recent Canadian Community Health Study [20]. ADHD children in this study consumed the same percentage (28%) of Low-Nutrient Density (LND) foods as reported in age-matched population normal data [33] which is important given that LND foods are known to displace nutrient-rich foods in the diet [33]. Maternal concerns expressed during the study that their ADHD children are “junk food junkies” do not seem to be empirically justified in this sample, in comparison to children of the same age and gender from the population. 

This is the first study to demonstrate significantly lower dietary intakes of micronutrients in ADHD children compared to population norms [44]. Despite the fact that children in this study were biased towards subjects of higher socioeconomic status, possibly contributing to subjects having greater access to the more costly Meat and Alternate foods known to be high in zinc, and copper, it is unknown why subjects consumed less Meat and Alternates than the norm.

This study is limited by the relatively small sample size and by lack of a specific normal control group. In addition, the study did not control for vitamin and mineral supplementation. That being said, all subjects had blood samples drawn in the morning in a nonfasted state and all analysis were performed at the same laboratory at BC Children's Hospital as the lab normal data with which the results were compared. 

This study adds to the growing literature demonstrating low serum zinc in ADHD children [8] and reports for the first time low dietary intake of zinc and copper and low serum copper status in a sample of ADHD children. 

Further research is needed to help identify the etiology, impact, and possible therapeutic implications of low micronutrient status in ADHD, given the essential nature of these micronutrients in the production of the neurotransmitters involved in ADHD.

## 5. Conclusion

This study establishes that the dietary intake of these ADHD children from Canada is comparable to local population norms in the intake of calories and of macronutrients; however, ADHD children in this study consumed significantly lower quantities of zinc and copper, but not iron. Half of the children in the sample consumed less than the recommended servings of Meat and Alternates, a major source of zinc and copper and a significant source of iron.

Since these ADHD children consumed the same percentage of Low-Nutrient-Density foods as children of the same age from the general population, it does not appear that displacement of trace minerals from excess “junk food” contributed to low dietary intakes of zinc and copper in these children.

Serum micronutrient status in this study adds support to a previous reports of low zinc [8] in ADHD children. In addition, this pilot study is the first to report that ADHD children may be at risk for low-serum copper status. 

The importance of these findings is that zinc, iron, and copper are essential cofactors in the production of dopamine and norepinephrine [12–14, 45, 46]; two neurotransmitters critical in the etiology of ADHD. Further research comparing dietary intake and nutrient status, controlling for medication and supplementation and including normal controls, is needed to determine if micronutrient deficiency is a cause, effect, or related to a third variable involved in ADHD.

## Figures and Tables

**Table 1:** Comparison of Macronutrient Distribution in the Diets of Children with ADHD with British Columbia aged 6–8 and 9–12 years.

Macronutrient	Mean ADHD*	Mean CCHS**	*P*-Value	95% CI of the Difference	
	(%)	(%)		Lower	Upper
Protein (%)					
6–8 years	14.08 ± 2.9	14.45	.596	−1.8204	1.0786
9–12 years	13.48 ± 2.5	14.23	.246	−2.0734	.5664

Carbohydrate (%)					
6–8 years	56.79 ± 9.2	55.63	.599	−3.4380	5.7625
9–12 years	53.49 ± 9.1	54.00	.827	−5.3626	4.3516

Fat (%)					
6–8 years	32.61 ± 8.1	29.91	.177	−1.328	6.7342
9–12 years	34.91 ± 6.6	31.76	.080	−.4222	6.7078

Energy (Kcal)					
6–8 years	1881 ± 377	2041	.090	−347.64	28.02
9–12 years	2476 ± 1042	2041	.081	59.45	931.01

*ADHD = Attention Deficit Hyperactivity Disorder

**CCHS = Canadian Community Health Survey data.

**Table 2:** Micronutrient intakes of children with ADHD compared to estimated average requirements (EAR).

Micronutrient	Mean Dietary Intake, mg/day	EAR^(1)^ mg/day	Meeting EAR (%)
Vitamin B_6_			
6–8 years	0.9 ± 0.5	0.5	89
9–12 years	1.18 ± 0.7	0.8	78

Iron			
6–8 years	12.6 ± 6.3	4.1	100
9–12 years	14.06 ± 32.3	5.9^(2)^/5.7^(3)^	100

Zinc			
6–8 years	6.44 ± 3.6	4.0	72
9–12 years	6.79 ± 2.8	7.0	39

Copper			
6–8 years	0.7 ± 0.4	0.34	72
9–12 years	0.8 ± 0.5	0.5	61

^(1)^EAR = Estimated Average Requirement Taken from the Dietary Reference Intakes [9]

^(2)^EAR for males aged 9–13 years

^(3)^EAR for females aged 9–13 years.

**Table 3:** Micronutrient intake data in ADHD children aged 6–12 years compared to population *d* *a* *t* *a* ^(1)^.

Micronutrient	ADHD	NHANESII^(2)^	*P*-value	95% CI of the Difference	
(mg/day)	(*n* = 36)	(*n* = 1581)		Lower	Upper
Vitamin B_6_	1.1 ± 0.5	1.7 ± 1.7	*P* < .001	−.8194	−.4829
Iron	17.2 + 2.34	14.1 ± 12.9	NS	−4.8052	11.0135
Zinc	6.6 + 3.2	10.0 ± 9.5	*P* < .001	−4.4683	−2.3001
Copper	0.7 + 0.4	1.1 ± 1.1	*P* < .001	−.5317	−2.447

^(1)^Values represent mean + SD.

^(2)^Population data derived from [34].

**Table 4:** Comparison of serum micronutrients of ADHD children with age-paired NHANES *I* *I* ^(1)^.

Serum Measure	Mean ADHD	Mean, NHANES II	*P*-value	95% CI of the Difference	
		[38]		Lower	Upper
zinc (*μ*mol/L)					
6–8 years*	11.1 + 1.6	12.9 + 2.9	*P* < .001	−2.463	−1.015
9–11 years**	11.2 + 1.5	13.6 + 2.2	*P* < .001	−3.163	1.612

ferritin (*μ*g/L)					
6–8 years	36.9 + 19.7	34.3 + 24.8	*P* = .582	−7.2207	12.5294
9–11 years	39.7 + 17.2	38.5 + 19.4	*P* = .786	−7.9672	10.3422

copper (*μ*mol/L)					
6–8 years	15.4 + 2.9	21.07 + 3.7	*P* < .001	−6.846	−4.278
9–11 years	15.4 + 2.9	19.73 + 3.5	*P* < .001	−5.562	−2.834

^(1)^Values represent mean + SD

*6–8 year old children *n* = 23

**9–11 year old children *n* = 16.
